# Primes and Consequences: A Systematic Review of Meritocracy in Intergroup Relations

**DOI:** 10.3389/fpsyg.2019.02007

**Published:** 2019-09-19

**Authors:** Ana Filipa Madeira, Rui Costa-Lopes, John F. Dovidio, Gonçalo Freitas, Mafalda F. Mascarenhas

**Affiliations:** ^1^Institute of Social Sciences, University of Lisbon, Lisbon, Portugal; ^2^Department of Psychology, Yale University, New Haven, CT, United States

**Keywords:** meritocracy beliefs, PWE, status legitimizing beliefs, priming, attitudes, behaviors, low-status groups

## Abstract

Psychological interest in Meritocracy as an important social norm regulating most of the western democratic societies has significantly increased over the years. However, the way Meritocracy has been conceptualized and operationalized in experimental studies has advanced in significant ways. As a result, a variety of paradigms arose to understand the social consequences of Meritocracy for intergroup relations; in particular, to understand the adverse consequences of Meritocracy for disadvantaged group members. The present research seeks to understand whether there is strong support for the idea that (manipulated) Meritocracy disproportionally affects members of low status groups, and also to understand which specific components of this norm have been successfully manipulated and to what consequences. And this is particularly important given the recent call for greater transparency in how the success of experimental manipulations is reported. Thus, we carried out a systematic review examining the content of different prime tasks, summarizing prime manipulation checks' effectiveness, and analyzing whether priming Meritocracy leads to less favorable orientations toward low status groups. Results across 33 studies revealed that despite the existing differences in the components highlighted, the salience of any of the Meritocracy dimensions facilitates the use of internal causal attributions, negative evaluations and stereotyping toward low status groups, affecting negatively decisions involving low-status group members, particularly in specific domains, as organizational contexts. These results carry both practical and theoretical implications for future research on the role of Meritocracy in intergroup settings.

## Introduction

Psychological interest in the belief of Meritocracy has significantly increased in the past 30 years. A social system is a Meritocracy when outcomes as wealth, jobs, and power are distributed on the basis of hard work, strong motivation, and personal ability (Kluegel and Smith, 1986; Jost and Banaji, 1994). Meritocracy beliefs are a psychological construct involving socially shared perceptions of a social system as meritocratic, which may or may not conform to the actual meritocratic nature of the system. A reason for the increasing interest in Meritocracy beliefs has to do with the merit-based rewarding system, very appealing among progressive societies, embodying a preference for social equity principles (Deutsch, 1975; Tyler, 2014). In fact, the practicing of rewarding good (or right) deeds is a symptom of a well-functioning society. Thus, the art of developing an incentive system based on the idea of merit has gained strength in the development of educational, organizational, and social policies, and has become an integral part of political discourse, particularly among western countries (e.g., Britain, the great Meritocracy: Prime Minister's speech, 2016).

In a psychological sense, Meritocracy beliefs constitute a worldview, or ideology, that broadly embraces the idea that equal opportunities exist, allowing upward social mobility (Feldman, 1983; Hochschild, 1996) in a way that individuals can change their economic and social circumstances (Taylor and Moghaddam, 1994). Economic and social success achieved is determined by internal factors, such as hard work, ability and individual responsibility, and not by privileged social relationships. Thus, individual merit, rather than social or power categories (Tajfel, 1978), determines individual success because any individual can improve their social status as long as they work hard, are motivated, and talented (Kluegel and Smith, 1986; Jost and Banaji, 1994). As such, Meritocracy beliefs have a deep impact on the way people think about and act toward low status groups. The current work aims to present a systematic review of the research that tested the influence of meritocracy beliefs on psychological and behavioral outcomes involving low status groups. Given our interest in research that demonstrates meritocracy as the cause of such outcomes, the current review focuses on those studies that have sought to experimentally activate meritocracy beliefs. Consequently, a complementary goal of this systematic review is to examine the content of these different meritocracy manipulations and the extent to which these different meritocracy primes have been successful^1^.

Before presenting the systematic review, the next section discusses different conceptualizations of meritocracy or similar concepts generally associated with it. By discussing the implications of these conceptualizations for intergroup relations, we further show the relevance of providing this systematic review. After that, we succinctly review correlational research showing how Meritocracy beliefs, traditionally construed as a central cultural value, have been increasingly associated with intolerance and dislike of members of low-status groups, and how this negative association can have systematic and important effects for intergroup relations. In the last section before presenting the methodological aspects of this review, we briefly review the types of prime paradigms typically used in socio-psychological research and how these can be (and have been) used to activate meritocracy beliefs.

### Conceptualizations of Meritocracy

The way in which the term Meritocracy is portrayed in the literature varies. One way is the conceptualization of Meritocracy beliefs as one among various ideologies that serve to maintain a status-based hierarchy (Major and Kaiser, 2017). In this conceptualization, the term status-legitimizing beliefs (SLBs) is used to describe how hard work and upward social mobility, components of Meritocracy, are used to interpret situations in ways that justify social inequalities (Jost et al., 2004; Jost and Hunyady, 2005). Thus, SLBs contain two out of the four Meritocracy beliefs core dimensions.

Another way is the Protestant Work Ethic belief (PWE; Weber, 1958). PWE reflects the belief that hard work leads to success, which as described earlier, is a core component of Meritocracy beliefs. Thus, PWE belief is a component of Meritocracy. Interestingly, both conceptualizations of Meritocracy belief seem to converge to the same *justifying* motivated reasoning (Kunda, 1990) through which low-status individuals are allegedly more likely to be discriminated against, and more likely to be held responsible for their relative disadvantage position (Levy et al., 2005, 2006, 2010; Major and Kaiser, 2017).

Despite these similarities, few attempts have been made to systematically integrate the findings of these two lines of research. Such integration could allow a theoretical unification that (a) incorporates Meritocracy, SLBs and PWE effects on socially relevant intergroup outcomes; (b) delimits the conditions under which different processes come into play within these concepts, and (c) clarifies which dimensions of each construct are being primed for the producing of various effects.

A potential theoretical unification is important because it allows a better understanding about what it means to endorse Meritocracy beliefs, as the two research lines mentioned earlier show an interesting pattern: Meritocracy beliefs may have dual implications for intergroup relations. One implication is that Meritocracy beliefs can operate as *social equalizer*, allowing people to achieve higher status, or a social *justifier* meaning (e.g., Levy et al., 2006), acting as a SLB by offering a socially acceptable explanation that stabilizes existing status differences. Whether Meritocracy beliefs acts as an equalizer or justifier depends on their correspondence with the actual dynamics of the social system. When a system is truly meritocratic, stronger mobility beliefs may help galvanize efforts among appropriately motivated and capable individuals for social mobility. However, when a system is not meritocratic but people believe that it is a meritocracy, members of low status groups may be inclined to see their social position as legitimate and thus be accepting, while high status group members may infer low status groups as individually responsible for their disadvantage position in the social system (McCoy and Major, 2007; Rüsch et al., 2010). The other, largely independent implication, is that Meritocracy beliefs can be descriptive, characterizing perceptions of the current social system, or prescriptive, providing a standard of what ought to be (Son Hing et al., 2011). For example, while descriptive Meritocracy—the belief that Meritocracy exists—is related to other legitimizing ideologies, such as political conservatism, racism, social dominance orientation, and right-wing authoritarianism, prescriptive Meritocracy—the belief that Meritocracy should exist—is argued to be unrelated to explicit and implicit negative attitudes toward low status groups (Son Hing et al., 2011).

### Meritocracy and Intergroup Attitudes and Behaviors

The former section explained how different conceptualizations of meritocracy can have different implications for intergroup relations. In this section we succinctly review correlational research showing how meritocracy is associated with intergroup attitudes and behaviors.

Research shows that the sense that one's position in society is based on one's own individual merit or hard work, is sufficient to deny the extent of societal inequality and, thus, overestimate current levels of economic equality. For example, the more individuals believe that Meritocracy exists, the more likely they are to deny economic inequalities and discrimination (e.g., Knowles and Lowery, 2012), and to overestimate racial equality (Kraus et al., 2019) and less likely to support for policies designed to reduce those inequalities (e.g., Son Hing et al., 2002; Garcia et al., 2005), at least under certain circumstances. Moreover, Meritocracy beliefs are strongly related to making internal attributions for the situation of disadvantaged groups, including women, people with mental illness, and less educated people (Major et al., 2002; Rüsch et al., 2010; Kuppens et al., 2018). Given that the low status groups are seen as particularly blameworthy for their own situation, this suggests a link between meritocracy and legitimization of inequality.

Among high status groups, the more individuals believe that Meritocracy exists, the more likely are to endorse positive stereotypes (e.g., intelligent, hardworking; Jost, 2001) and to deny White privilege (Phillips and Lowery, 2015). Conversely, believing that Meritocracy exists is related with greater negative internal attributions for the relative disadvantage position of low status groups (Haney and Hurtado, 1994; Fraser and Kick, 2000). Among low status groups, research finds a positive relationship between endorsing meritocracy and a greater sense of control (McCoy et al., 2013); yet, in the long run it is associated with lower self-esteem, self-blame, and depression (Major et al., 2007), and also higher blood pressure (Eliezer et al., 2011), particularly when low status targets face discrimination.

Additionally, a meta-analysis shows a significant positive relationship between Meritocracy beliefs (e.g., PWE) with both prejudice and policy attitudes, particularly in Western societies (Rosenthal et al., 2011). The research described above points to the belief in Meritocracy as the potential cause of negative outcomes toward low status groups. Experimental attempts to demonstrate this idea have amounted to a significant body of research in the last 20–30 years. Focusing on these experimental manipulations of meritocracy allows us not only to make informed statements about causality but also to contribute to the discussion about the contextual salience of these types of norms and its impact on attitudes and behaviors. Virtually, all the manipulations employed in the experimental research to be reviewed attempted to mentally activate content related with meritocracy and/or associated concepts^2^. Therefore, before describing the systematic review process, the next section presents the two fundamental types of priming and recapitulates the reasons and goals of this systematic review.

### Types of Meritocracy Primes

The ability to temporarily activate Meritocracy beliefs has been used by researchers investigating the causal role Meritocracy plays in intergroup processes. One way to activate Meritocracy is through priming tasks encoding cues that are relevant to the construct, providing temporary access to the mental content of meritocracy beliefs. A prime can directly (explicitly) or indirectly (implicitly) activate meritocracy beliefs, and people may be conscious of this activation (explicit impact) or unaware of the thoughts, feelings, beliefs, and attitudes that have been activated (implicit impact). Once the construct is activated in memory, it is likely to be used as a basis for subsequent judgments (Higgins et al., 1977; Srull and Wyer, 1979) and to influence behavior (Bargh, 1989). The activation or implicit priming tasks are currently under intense experimental scrutiny and controversy (Schimmack et al., 2017) but the evidence does show that priming occurs, at least with some temporary influence (Weingarten et al., 2016).

The typical explicit priming paradigms present subjects with stimuli or instructions that are explicitly in association with cues that are relevant to the construct. This happens because individuals have explicit access to their belief system. In such paradigms, individuals typically read a brief article or are asked to report their level of endorsement of a given belief or attitude. This type of explicit priming paradigms increase the availability of the mental content (e.g., attitudes or beliefs) storage in memory, promoting the creation of cognitively consistent inferences (Schuman and Presser, 1981; Bradburn, 1982).

The implicit priming paradigms, in turn, seek to indirectly activate the belief by making participants engage in a task where the concept is activated outside the individual's consciousness (Greenwald and Banaji, 1995). In such studies, participants are presented with words related to the construct in a camouflaged manner (e.g., Srull and Wyer, 1979). A good example of an implicit priming is the unscramble sentence task, where participants are asked to perform a task, where they have to unscramble a set of 5 words into a 4-word meaningful sentence (Srull and Wyer, 1979; McCoy and Major, 2007). In this case, the salience of Meritocracy beliefs is sought to occur when people temporarily view the world through the lens of this belief system, because it is storage in their minds. As a result, if the activation succeed is sought to be reflected in individual's endorsement of the belief in Meritocracy, in that primed individuals should express a higher agreement with the belief, compared to the control condition.

The way the priming effectiveness has been assessed differs as a function of the type of task used to temporarily activate Meritocracy beliefs. While some studies measure the salience of meritocracy (Redersdorff et al., 2016), others measure the extent to which subjects endorse meritocracy beliefs (Levy et al., 2006; Castilla and Benard, 2010; Darnon et al., 2017). Consequently, the heterogeneity described both in the nature of the prime and in the forms that activation and the manipulation checks can assume, makes it challenging for researchers to ascertain the best content for meritocracy activation, and the best practices for implementing manipulation checks (Hauser et al., 2018).

Given the diffuseness of the current literature in this area and the focus on the different types of approaches and assumptions made about Meritocracy across studies, we pursued a systematic review of the literature (and not a more focused meta-analysis of particular findings). Specifically, as stated above, the main goal of the current review is to summarize the research on the impact of Meritocracy priming on psychological and behavioral outcomes for low status groups. As a secondary related goal, we summarize the content of the different prime tasks used in these studies, and review to what extent the prime succeeded at activating the socio-psychological construct.

## Methods

### Search Strategy

For this systematic search, conducted in 2018, we developed a search strategy using a combination of PICOS and SPIDER tool (Cooke et al., 2012). This search strategy was tailored to four databases: Scopus, PsycINFO, EBSCO, Web of Science, and the search terms used were the following: Meritocr^*^ OR ideology OR “system justification theory” OR “social mobility” OR “Protestant work Ethic” OR individualism OR “belief in a just world” OR authoritarianism AND “racial attitudes” OR “social attitudes” OR “political attitudes” OR “implicit attitudes” OR evaluation OR belief OR perception OR “decision making” OR “behavioral intentions.” All searches spanned from database inception until 2018, and included journal articles and academic dissertations (Master's and Ph.D.), published in English, Spanish, French, and Portuguese. Beyond database search, we used direct-to-researcher channels (e.g., servers list), as recommended by Cooper et al. (2009).

### Selection Criteria

The selection criteria were based on the PRISMA Statement (Moher et al., 2009). The phenomena of interest in the criteria of inclusion included any experiment using Meritocracy as an independent variable and any outcome on explicit and/or implicit attitudes, racial, social and political evaluations, perceptions, beliefs, and decision-making involving members of low status groups.

At the initial screening stage, two reviewers judged the title and abstracts against the inclusion criteria. Both reviewers read the title and abstract and applied the inclusion/exclusion criteria from the screening form to make a decision on whether or not to include the study in the review. The decision for inclusion vs. exclusion on the study was recorded in a screening form (i.e., Screening Titles and Abstracts online form). If the title and the abstract met the inclusion criteria then the full-text copies of all studies were retrieved for the next screening level.

At the second level of screening, two authors reviewed the full-text articles independently for the relevance of research aim. A web-based software was used to partially automate the screening process (Covidence, systematic review software)^3^. Any disagreements were resolved via discussion.

Eighty-eight empirical articles were assessed for full-text eligibility. Sixty-five out of the 88 articles were excluded because were correlational (*N* = 23), did not experimentally manipulate any Meritocracy-based construct (*N* = 16), did not measure attitudes or decisions toward low status groups (*N* = 11), were not quantitative (i.e., systematic/literature reviews, case studies; *N* = 8), were conference proceedings, newspapers articles (*N* = 6), and one was not available (*N* = 1).

**Figure 1:**
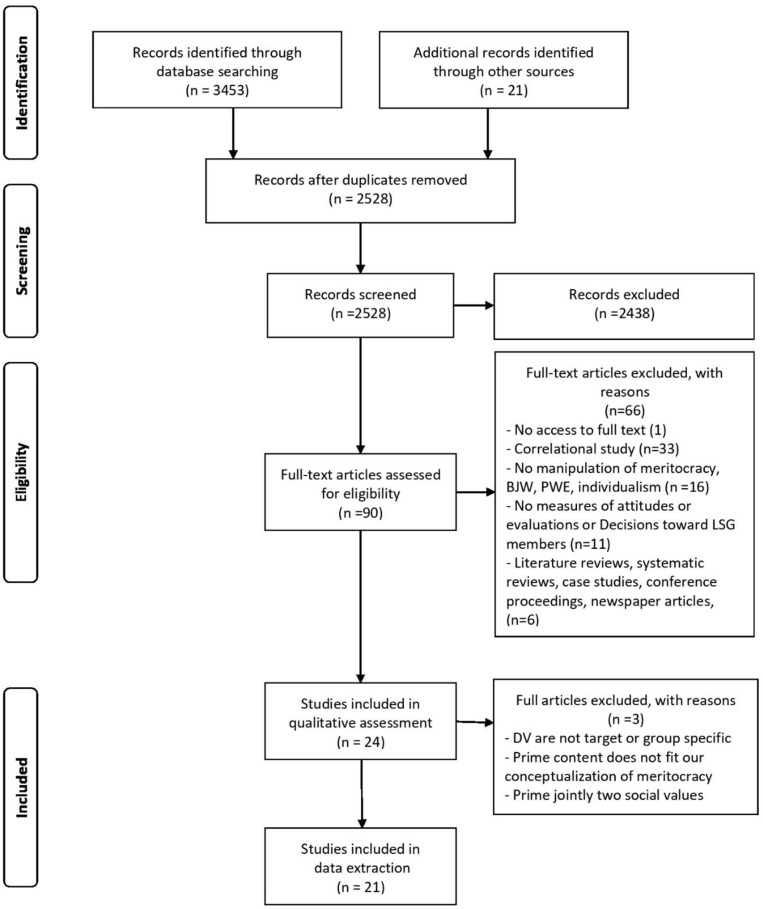
PRISMA flow diagram of search results (Moher et al., 2009).

Thus, a total of 23 articles were identified that met the inclusion criteria. A PRISMA flow diagram (Figure 1) summarizes the information on the phases of the systematic review process.

### Quality Assessment

To assess the quality of the articles we created a coding sheet attempted to assess the likely internal, construct, and statistical validity of the inferences arising from the studies (Table S4). We approach this aspect of the review creating a coding sheet based on the framework provided by Valentine and Cooper (2008). The coding sheet included characteristics at the study level and at the outcome level; addressed the internal validity (e.g., to what extent the procedure permits an ambiguous conclusion about the experimental manipulation effectiveness), construct validity (e.g., to what extent participants were treated and the outcomes measured in a way that is consistent with the definition of the paradigm and its proposed effects), and statistical validity (e.g., to what extent accurate estimates could be derived from the study report; see Tables S3, S4).

### Data Extraction

In the data extraction phase, 21 articles were selected and the characteristics extracted were:

1. Characteristics of study and participants: total sample size; the number of participants per group; mean age and standard deviation; participant's sex; the number of experimental and control groups; the number of independent variables, type of design.
2. Characteristics of the outcome measures: assessment (implicit or explicit); dimension (perceptual; attitudinal; behavioral); toward the self or others; source (original or adapted).
3. Characteristics of experimental manipulation: experimental group content (e.g., Meritocracy, PWE, social mobility); control group content, source of the manipulation (original or adapted).
4. Estimation of effect sizes: number of participants per group, descriptive statistics (mean and standard deviations for each group), and student's *t*, when M and *SD* and *N* per group were not fully reported.

### Data Analysis

#### Prime Content

Prime description and dimensions of Meritocracy were identified in each study. Using a Likert scale, from 1 = Not at All to 4 = Fully, three authors analyzed to what extent the four conceptual dimensions were reflected in the prime: two personal dimensions (e.g., effort and internal control) and two structural dimensions (e.g., social mobility and equal opportunities). As to the two personal components, *Effort* reflects the idea that societal rewards are based on effort and ability and *internal control* reflects the idea that people have control over their own success and failures. As to the structural components, *social mobility* reflects the idea that people can achieve success and *equal opportunities* reflect the idea that society/organizations provide equal opportunities for all.

### Calculating the Effect Size

#### Prime Effectiveness

For studies reporting a sample size per group, mean, and standard deviation, we used a spreadsheet to estimate the size of the effect of each measure (Lakens, 2013). We calculated the effect sizes using Cohen's *d* (Cohen, 1988).

#### Intergroup Outcomes

Studies with a single experimental factor and a single continuous outcome variable reporting sufficient information, effect sizes for the Meritocracy were calculated by subtracting the control group mean from the experimental condition (i.e., Meritocracy) group mean and dividing the result by the pooled standard deviation. Studies with a single experimental factor and two continuous outcome variables (e.g., low status and high-status means) reporting sufficient information, Effect sizes for social status were calculated by subtracting the high status mean from the low status mean and dividing the result by the pooled standard deviation, at each level of the experimental factor. Studies with two experimental factors and a single continuous outcome variable reporting sufficient information, effect sizes were calculated by subtracting the control group mean from the experimental condition (i.e., Meritocracy) group mean and dividing the result by the pooled standard deviation, at each level of the second experimental factor.

## Results

### Study Selection and Characteristics

All available data from experiments was summarized in an excel spreadsheet to the fullest extent possible. Data were entered by two reviewers and independently checked by another reviewer. As a result of data unavailability, and heterogeneity in experimental designs, we determined that a statistical meta-analysis would be inappropriate. Instead we present whenever possible effects sizes of individual studies and provide a narrative synthesis. We were unable to test for publication bias owing to limitations on data.

Of the 33 selected experiments, the majority are published manuscripts, including 28 journal articles, one working paper and one chapter. The remaining three are unpublished manuscripts (e.g., master and doctoral thesis). The research on the topic of Meritocracy had focused extensively on the United States, while the remaining research is distributed among France (*N* = 3), the Netherlands (*N* = 2), and Portugal (*N* = 3). Fourteen experiments were conducted within an applied field. The majority were conducted in an organizational context (e.g., Castilla and Benard, 2010), two studies took place in the educational context (Darnon et al., 2017, 2018), two others were aggregated into a health-related domain (Quinn and Crocker, 1999; Newsom, 2014), two experiments were conducted within moral dilemmas scenarios (Moreira, 2016) and one in a social domain (Levy et al., 2006). The remainder experiments were unspecified domain-wise.

Participants were reportedly surveyed in the Lab (*N* = 11), online (*N* = 9), or in the classroom/at campus/school (*N* = 7), and one in the street; the remainder (*N* = 5) did not report where the experiment was carried out. The majority of the samples consisted of adults (*N* = 31). All studies used an experimental design, where 28 were between-subjects and 5 a mixed design.

A large portion of studies involve a female target (*N* = 13), immigrants (*N* = 3), socioeconomic status (*N* = 4), ethno-racial groups (*N* = 3), one addresses mental illness stigma (Table S2).

#### Manipulation Characteristics

Of the 33 manipulations, 22 used an explicit prime (e.g., reading a text; completing a scale) and 11 use an implicit prime task (e.g., unscrambling words). A large portion of primes reported Meritocracy (*N* = 21) as the theoretical construct, while six studies report Protestant Work Ethic and three report perceptions of success or social mobility. A single prime uses levels of prescriptive Meritocracy (moderated vs. high), six focused on descriptive meritocracy, six reported a mixed procedure combining prescriptive and descriptive meritocracy, and four primes could not be specified (see Table 1).

**Table 1:** Summary of study characteristics and construct salience (MS) results and effect sizes.

**Study**	**Sample**	**Type meritocracy**	**Manip group**	**Control group**	**Second IV**	**DV**	**Results**	**Effect size (95% CI)**	
**Meritocracy**										
Castilla and Benard (2010)[study 1]	♀ (64) ♂ (163) M_age_ = 29.71 (3.89)	Both	Meritocracy -based evaluation	Regularity of evaluation	None	Meritocracy	> endorsement of Meritocracy in the Meritocracy condition, vs. control	0.35 [0.09, 0.61]	
Chatard et al. (2006)	♀ (24) ♂ (31) M_age_ = 39.17 (NR)	Prescriptive	“Moderated” Meritocracy	“Radical” Meritocracy	Positive Discrimination (equivalent level vs. minimum required vs. unconditional preference)	Meritocracy	> endorsement of Meritocracy in the moderated merit condition condition, vs. radical merit condition.	0.69 [0.22, 1.16]	
Darnon et al. (2017)	♀ (80) ♂ (66) 3(unspecified) M_age_ = 10.13 (.51)	Descriptive	Meritocracy	Neutraltext about frogs' ability to anticipate disasters	None	Belief School Meritocracy	> of Meritocracy in the Meritocracy condition, vs. control condition	−0.03 [−0.29, 0.23]	
Darnon et al. (2018)	♀ (68) ♂ (158) M_age_ = 40.15 (6.73)	Descriptive	Meritocracy	Neutraltext about backyard's features determines children's games		Belief School Meritocracy	= level of Meritocracy endorsement across both conditions	0.49 [0.23, 0.76]	
McCoy and Major (2007) [pilot study]	♀ (13) ♂ (19) M_age_ = 19.56 (1.58)	Both	Meritocracy	Neutral		Individual Mobility	> of social mobility in the Meritocracy condition, vs. control condition.	0.77 [ 0.05, 1.49]	
Laurin et al. (2011)	♀ (67) ♂ (24) M_age_ = 18.8 (NR)	Both	Meritocracy	Neutral	None	Societal fairness beliefs	> fairness societal belief in the Meritocracy condition than control condition	0.65 [0.08, 1.23]	
Redersdorff et al. (2016) Pilot study	♀ (34) M_age_ = 32.32 (6.29)	Unspecified	Meritocracy	Social equality	group composition (all male vs. gender-balanced)	Number of identified merit-related words.	> percentage of words related to Meritocracy identified in the Meritocracy cond vs. social equality cond.	*Not estimable*	
Thomson (2015) Pilot study	♀ (37) ♂ (17) M_age_ = 24.3 (NR)	Both	Meritocracy	Seniority	None	Correct identification of the compensation system as “merit-pay” or a “seniority-based pay.”	25/27 identified correctly the Company's Core values as meritocratic in the Meritocracy condition. 18/25 identified correctly the Company's Core values as non-meritocratic in the control condition.	*Not estimable*	
Pereira et al. (2009)	80 ♀ (49%) ♂ (51%)	Both	Meritocracy + Protestant Work Ethic (Katz and Hass, 1988)	Egalitarianism + Egalitarianism (Katz and Hass, 1988)	humanity: humanization vs. infra-humanization	Grades difference in the evaluation of two students solving a math's problem; higher scores indicated greater application of the meritocratic norm compared to the egalitarian norm.	> Application of the meritocratic norm in the meritocratic norm condition than in the egalitarian condition.	0.35 [−0.09, 0.80]	
**Protestant work ethic**										
Levy et al. (2006) [study 2]	Older group: ♀ (130) ♂ (39) (*M _*age*_* = 21.31). middle group: ♀ (106) ♂ (49) (*M_*age*_* = 14.99). youngest group: ♀ (40) ♂ 41 40 females; *M_*age*_* = 10.80).	Descriptive	Protestant work ethic	Anti–protestant work ethic		Protestant ethic work	> endorsement of PWE in the Pro – PWE condition	Older, Cohen's *d* = 0.75 Middle, Cohen's *d =* 0.73 Youngest, Cohen's *d =* 1.37	
Levy et al. (2006) [study 3]	♀ (63) ♂ (72) (*M_*age*_* = 21.45).	Descriptive	Protestant work ethic	No task	task instructions: Justification vs. Definition	Protestant Ethic Work	= level of Meritocracy endorsement in the Meritocracy justification (*vs*. definition Condition)	PWE 0.04 [−0.45, 0.53]	No Task *No estimable*
Newsom (2014)	♀ (201) ♂ (71) M_age_ not reported	Descriptive	Protestant work ethic message	Inclusive message		Protestant ethic work	= level of PWE endorsement across both cond.	0.13 [−0.11, 0.37]	
Biernat et al. (1996) [study 2]	185 White *Sex and age unreported*.	Both	Protestant Work Ethic Speech	Egalitarianism Speech	value: violation vs. support	Opinion on how to cut funding on two minority status organizations vs. academic honors societies.	For minority org: > funding cut on the PWE (vs. EG cond) Honors society: = funding cut	Minority org, Cohen's *d =* 0.29 Honors society: *Not estimable*	
Quinn and Crocker (1999)	♀ (118) Normal weight (6) Overweight (59)	Descriptive	Protestant Work Ethic Message	Inclusive Message	all participants read a text about social devaluation of being overweight	one-sentence summary of the prime;powerfulness of the ideology primes;political orientation.	= level of powerfulness in the Protestant ethic and the message and the inclusive. No differences in polit. orientat measure	*Not estimable*	
Ho et al. (2002) [study 1]	97 participants *Sex and age unreported*.	Unspecified	Perceived Economic Success	CG 1—Animal Video Control conditionCG 2—No Video Control condition	None	Opportunity and Social Mobility in the US	> belief in Social Mobility in the US in the economic success condition (vs. Control)	0.44 [0.04, 0.84]	
Ho et al. (2002) [study 2]	43 participants *Sex and age unreported*.	Unspecified	description of the “success” of Asian Americans	No description of the “success” of Asian Americans	None	Opportunity and Social Mobility in the US	> belief in Social Mobility in the US in the description success condition (vs. Control)	Hedges gs = 0.43	
Ryan et al. (2012)	♀ (137) ♂ (96) M_age_ not reported	Unspecified	High Social Mobility	Low Social Mobility	Group gender composition: all male vs. balanced composition	subjective tokenism:		*Not reported*	

### Synthesized Findings

#### Meritocracy Activation

A detailed overview of the Meritocracy Activation (MA) is depicted in Table S1. Five multi- experiments present an original prime aiming to activate aspects of the Meritocracy construct (Chatard et al., 2006; McCoy and Major, 2007; Pereira et al., 2009; Castilla and Benard, 2010; Redersdorff et al., 2016; Darnon et al., 2017, 2018), four primes focused on Protestant Work Ethic (Katz and Hass, 1988; Biernat et al., 1996; Quinn and Crocker, 1999; Levy et al., 2006) and two primes focused on perceptions of success and social mobility (Ho et al., 2002; Ryan et al., 2012). The remaining used either the same and modified version of the original or one of the primes mentioned above.

Within Meritocracy construct, all incorporate, to a large extent, the effort/hard work and internal control dimension of the value. Additionally, a few captures, to a large extent, the social mobility aspect (e.g., McCoy and Major, 2007).

Within the PWE construct, all focus the effort and hard work aspect of this value. Additionally, three of them capture, to some extent, the *internal control* aspect (Katz and Hass, 1988; Biernat et al., 1996; Quinn and Crocker, 1999).

As expected within the social mobility construct, the studies focus specifically on this structural component of Meritocracy ideology. A prime focused on social mobility beliefs, associated with tokenism (Ryan et al., 2012). Another prime aiming to manipulate perceptions of success and social mobility presented a video of a program showing several award winners' bleak beginnings, the obstacles that they had to overcome, and the qualities that they possessed that enabled them to succeed, therefore capturing the idea of hard work and internal control alongside the idea that anyone can move upward in the social ladder (Ho et al., 2002, *study 1*).

The majority of the tasks uses explicit Meritocracy prime. The explicit activation of Meritocracy is made via reading a company's core values (Castilla and Benard, 2010; Thomson, 2015), filling out a questionnaire (Chatard et al., 2006), via a comprehension text task (Costa-Lopes et al., 2017 study 1; Pereira et al., 2009) or via a task where participants are instructed to put six events of meritorious people on a historic timeline ranging from 1900 to 2013 (Redersdorff et al., 2016). The explicit activation of PWE is made via reading a political speech (Quinn and Crocker, 1999; Newsom, 2014) or reading a newspaper report concluding that “people who work hard do well and have a successful life” (Levy et al., 2006) or listening to an audiotaped speech (Biernat et al., 1996).

A subtle prime consists of using a scrambled sentence task to prime Meritocracy (McCoy and Major, 2007). Studies using this type of prime share the same procedure: participants are given 5 min to unscramble 20 sets of 5 words into 4-word sentences. The prime sentences focus mainly on two aspects of the Meritocracy value—the idea that societal rewards are based on effort and ability (e.g., “Effort leads to prosperity”) and on the idea that people have control over their own success and failures (e.g., “responsible people get ahead”). And, to some extent, the prime focuses on the “social mobility” belief (“earn a good living”).

#### Priming Effectiveness

Of the total of studies, 17 do not have a prime manipulation check, 2 use a measure of how much the concept is salient (Thomson, 2015; Redersdorff et al., 2016), 2 studies include a measure to quantify how much the concept was applied in a subsequent outcome (Biernat et al., 1996; Pereira et al., 2009, study 2), and one study included a measure of the powerfulness of the concept. The remaining allow the estimation of prime affecting the concept endorsement (see Table 1).

##### Meritocracy prime

As depicted in Table 1, three studies assessing manipulation's effectiveness show the priming condition affecting Meritocracy endorsement (Chatard et al., 2006; Castilla and Benard, 2010; Darnon et al., 2017), while two studies reported no significant differences between conditions (Newsom, 2014; Darnon et al., 2018). The outcome of interest varies across studies: four studies measured the belief in meritocracy (Chatard et al., 2006; Castilla and Benard, 2010; Darnon et al., 2017, 2018), two studies using the same implicit prime, reported different measures for assessing its effectiveness, namely (a) perception of individual mobility and (b) societal fairness (McCoy and Major, 2007; Laurin et al., 2011).

##### Protestant ethic work prime

Studies assessing a prime manipulation check vary in terms of the outcome of interest. Among those who reported an MC, in three studies PWE scale was used as a prime MC, while two use other forms of checking [e.g., (1) to give an opinion on how to cut funding on two minority status organizations vs. academic honors societies, and (2) to rate the powerfulness of the ideology prime].

Only in one of the three studies using PWE scale as a prime MC, participants strongly endorsed PWE to a greater extent than did participants in the control group [(Levy et al., 2006), study 2]. In the remaining two, PWE endorsement was not affected by the priming task (Levy et al., 2006, study 3; Newsom, 2014).

##### Economic success and social mobility prime

Participants who were primed solely with the perception of economic success or primed about the economic success of a specific group (e.g., Asian Americans) perceived opportunity and social mobility in the United States to be significantly greater than participants in the control group (Ho et al., 2002).

#### Impact of Meritocracy Prime on Outcomes Toward Low Status Groups

##### Does Meritocracy predict less favorable Intergroup Attitudes?

*Explicit and Implicit Prejudice* The results presented in Table 2 show that priming participants with Meritocracy or PWE increases levels of both implicit prejudice toward immigrants (Costa-Lopes et al., 2017) and explicit racial prejudice and decreases levels of positive racial attitudes (Katz and Hass, 1988). Interestingly, priming participants with prescriptive *moderated* Meritocracy increased levels of negative attitudes toward women (e.g., sexism) compared to priming participants with prescriptive *radical* Meritocracy (Chatard et al., 2006).

**Table 2:** Summary of studies related with the Impact of Meritocracy on Attitudes, Beliefs and Perceptions involving Low status groups.

**Study**	**Prime construct**	**Outcome**	**Results**	**Effect size (95% CI)**
Chatard et al. (2006)	Meritocracy	Sexism	Participants in the moderated Meritocracy prime scored higher than participants in the radical Meritocracy prime.	*d*_s_ = 0.60 [0.04, 1.16]
Costa-Lopes et al. (2017) [study 1]	Meritocracy	Implicit attitudes	Participants in the Meritocracy prime scored higher than participants in the control condition.	*d*_s_ = 0.61 [ −0.03, 1.24]
Costa-Lopes et al. (2017) [study 2]	Meritocracy	Implicit attitudes	Individuals' level of implicit prejudice at Time 2 increased in the Meritocracy prime, but not in the control group.	*d_*z*_*_Meritocracy_ = 0.55*d_*z*_*_control_ = −0.02
Katz and Hass (1988)	PWE	Pro-black attitudes	Participants in the PWE prime score lower than participants in the Egalitarianism prime.	*d*_s_ = −0.52 [ −1.16, 0.12]
		Anti-black attitudes	Participants in the PWE prime score higher than participants in the Egalitarianism prime.	*d*_s_ = 0.76 [0.11, 1.42]
Ho et al. (2002) [study 1]	Meritocracy + Economic success	Negative stereotypes toward blacks	Participants in the prime condition score higher than participants in the control condition.	*d*_s_ = 0.40 [0.00, 0.80]
		Perceived racial discrimination	Participants in the prime condition do not perceive significantly less racial discrimination than participants in the control condition.	*Not estimable*
Ho et al. (2002) [study 2]	Intergroup comparison of Perceived Success	Negative Stereotypes toward Mexicans	Participants in the prime condition score higher than participants in the control condition.	*d*_s_ = 0.75 [0.12, 1.38]
		Internal Attributions for the low status position	Participants in the prime condition score higher than participants in the control condition.	*d*_s_ = 0.68 [0.05, 1.30]
		Attributions of lower status position	Participants in the prime condition score higher than participants in the control condition.	*d*_s_ = 0.64 [0.02, 1.26]
		Perceived racial discrimination	Participants in the prime condition do not perceive significantly less racial discrimination than participants in the control condition.	*Not estimable*
Pereira et al. (2009)	Meritocracy	Discrimination: opposition to Turkish Adhesion to EU	Participants in the Meritocracy prime show a higher opposition than participants in the control condition.	*d*_s_ = 0.39 [−0.23, 1.02]

*Stereotyping* Low status groups such as African Americans and Mexicans were portrayed less favorably in the prime condition, than in the control conditions. Specifically, participants in the prime condition were more willing to infer negative internal attributions for African and Mexican Americans, by agreeing that often they lack the values that are needed for social advancement or that many lack the motivation or willpower that is necessary for economic success (Ho et al., 2002).

##### Does Meritocracy predict opposition to equality between groups?

*Egalitarianism* As seen in Table 3, levels of Egalitarianism were found to decrease after the PWE prime, but only in participants who were instructed to use PWE as an *argument* to justify socioeconomic status quo inequality, as opposed to thinking about the *meaning* of PWE [(Levy et al., 2006), *study 3*]. Moreover, PWE effects on egalitarianism endorsement were found to be moderated by age. While in children aging 10–12 and 14–16 years old levels of Egalitarianism increased in the PWE-prime condition (vs. control), among young adults (18–25 years) levels of Egalitarianism were found to decrease after the PWE prime (Levy et al., 2006, *study 2*).

**Table 3:** Summary of studies related with the Impact of Meritocracy and Equality between groups.

**Study**	**Prime**	**Target**	**Outcome**	**Moderator**	**Results**	**Effect Size**	
						**Prime**	**Control**
Chatard et al. (2006)	Meritocracy	F	Support for AA	Type of Positive Discrimination PolicyEquivalent level	Participants in the *strong* Meritocracy prime, were less oppose to affirmative action, than participants in the *moderate* Meritocracy prime.	*Not estimable*	
				Minimum required	Participants in the *strong* Meritocracy prime, were less oppose to affirmative action, than participants in the *moderate* Meritocracy prime.		
				Unconditional preference	Participants in the *strong* Meritocracy prime, were strongly more oppose to this affirmative action policy, than participants in the *moderate* Meritocracy prime.		
Darnon et al. (2018)	Meritocracy	None	Interest in the equalizing pedagogical method	Equalizing method	Participants in the Meritocracy prime did not scored significantly different from participants in the control condition.	*d_*s*_* = 0.14	
				Enhancing method	Participants in the Meritocracy prime did not scored significantly different from participants in the control condition.	*d_*s*_* = −0.16	
			Behavioral engagement in the equalizing pedagogical method	Equalizing method	Participants in the Meritocracy prime did not scored significantly different from participants in the control condition.	*d_*s*_* = 0.22	
				Enhancing method	Participants in the Meritocracy prime did not scored significantly different from participants in the control condition.	*d_*s*_* = −0.01	
Levy et al. (2006) [study 2]	PWE	None	Egalitarianism	Age	**10–12 years**. Participants in the prime condition reported higher levels of egalitarianism than participant in the control condition	*d_*s*_* = 0.52	
					**14–16 years**. Participants in the prime condition reported higher levels of egalitarianism than participant in the control condition	*d_*s*_* = 0.43	
					**18–25 years**. Participants in the prime condition reported lower levels of egalitarianism than participant in the control condition	*d_*s*_* = −0.34	
Levy et al. (2006) [study 3]	PWE	None	Egalitarianism	task instructions (justification vs. definition) × task content (PWE vs. control)	Participants in the Justification condition reported lower levels of egalitarianism than participant in the definition condition.	Task Content: PWE *D_*g*_* = 0.77	
					Participants in the Justification condition did not scored differently from participant in the definition condition	Task Content: Control *d_*g*_* = 0.04	
Wellman et al. (2016)	Meritocracy	None	Support for AA	*None*	Participants in the Meritocracy prime show less support for Affirmative actions compared to the control condition.	*D*_g_ = −0.35 [0.71, 0.01]	
			Zero-sum beliefs		Participants in the Meritocracy prime endorse zero-sum beliefs more compared to the control condition.	*d*_s_ = 0.37 [0.01, 0.73]	

*Promotion of equality in school* Interest and Behavioral engagement in a more equalizing pedagogical method in school was found to be similar among participants exposed to the meritocracy condition and the control condition (Darnon et al., 2018).

*Opposition to Positive Discrimination Policies* Opposition to Affirmative Action policies increased in the Meritocracy condition compared to control, along with a higher endorsement of Anti-White bias beliefs (e.g., the idea that efforts to reduce discrimination against minorities have led to increased discrimination against White people; Wellman et al., 2016). Another study found that opposition to Affirmative Action policies in the workplace varies as a function of the level of prescriptive Meritocracy and the type of policy (Chatard et al., 2006). Specifically, participants in the strong Meritocracy prime (vs. moderate Meritocracy prime) were more in favor of a positive discrimination policy when the policy was to hire a female candidate when (a) both female and male candidate have the same level of qualification or (b) when female's qualifications meet the minimum required for the position. No differences between strong vs. moderate prime conditions were found for the unconditional preference policy (e.g., the female candidate should be preferred).

##### Does priming Meritocracy beliefs lead people to make concessions as a function of the a) source or b) the target of the discrimination?

*Priming the Locus of Causality of the discriminated Low-Status target* As described in Table 5*, two* studies found that when the discriminatory behavior is attributed to discrimination, female participants exposed to the prime (vs. control condition), perceived less prejudice against the female candidate, endorsed gender stereotypes to a significantly higher degree (McCoy and Major, 2007) and judged the female target as less competent (Redersdorff et al., 2016). Interestingly, when the discriminatory behavior is attributed to internal factors (e.g., less competence), the discriminated female target is seen as more competent in the prime condition (vs. control condition), and is held equally responsible for the negative outcome across the two conditions (i.e., social equality and Meritocracy).

Moreover, female participants in the Meritocracy condition perceived the victim as more responsible when the negative outcome was attributed to her abilities and not to sexism. However, in the control condition, the same pattern did not occur, as the victim was perceived equally accountable, regardless of the locus of causality presented to female participants (Redersdorff et al., 2016).

*Priming discrimination against a High-Status target* Two studies found that exposing participants to Anti-male Bias predicts differentials for a low and high-status target. Perceiving Anti-male Bias in the prime condition (vs. control) increases positive evaluations and helping intentions toward a White male target (Wilkins et al., 2013). Interestingly, the opposite effect happens when the target is female: perceiving Anti-male Bias in the prime condition (vs. control) decreases positive evaluations and helping intentions toward a female target (Wilkins et al., 2018).

##### Does Meritocracy beliefs predict less favorable Evaluations of low-status targets?

*Competence* The relationship between the prime and perceptions of low-status targets' competence was found to be moderated by individuals' levels of PWE. One study shows that, when PWE is high, the Black Target is judged as less competent that the White target in the prime condition. While when PWE is low, the Black target is judged as equally competent as the White target in the prime condition (Biernat et al., 1996; see Table 4). In another study, the evaluation of the low-status target (e.g., female target) competence was found to be moderated by the causality (sexism vs. internal attributions) of discriminatory behavior in the workplace. When the discriminatory behavior is attributed to sexism, the discriminated female target is judged less competent in the prime condition (vs. social equality condition). Surprisingly, when the discriminatory behavior is internally attributed (e.g., less ability), the discriminated female target is more competent in the prime condition (vs. social equality condition; Redersdorff et al., 2016).

**Table 4:** Summary of studies related with Moderators of the relationship between Meritocracy and Intergroup Attitudes and Behaviors.

**Study**	**Prime**	**Target**	**Outcome**	**Moderator 2**	**Moderator 3**	**Results**	**Effect sizes**	
							**Prime**	**Control**
Biernat et al. (1996)	PWE	Black	Competence	Target status	HIGH PWE Endorsement	When PWE is high, the Black Target is judge as less competent that White target in the prime condition. When PWE is high, the Black Target is judge as equally competent that the White target in the egalitarian condition.	*Not estimable*	
					LOW PWE Endorsement	When PWE is low, the Black Target is judge as equally competent that White target in the prime condition. When PWE is low, the Black Target is judge more competent that White target in the egalitarian condition.	*Not estimable*	
			Social distance		HIGH PWE endorsement	When PWE is high, in the prime condition the Black Target is judge less favorably than White target. When PWE is high, in the egalitarian condition there's no differences between targets	*Not estimable*	
					LOW PWE endorsement	When PWE is low, in the prime condition there's no differences between targets. When PWE is low, in the egalitarian condition the Black Target is judge more favorably than White target.	*Not estimable*	
**Manipulation of meritocracy as a justifier belief**								
Levy et al. (2006) [study 4]	PWE	H	Monetary donation	Task instructions: Justification vs. Definition		Prime Condition. Participants in the, justification-condition participantsdonated significantly less money than definition-conditionparticipants Control Condition. The definition and control conditions did not significantly differ from one another.	*d_*s*_* = −0.62 [−1.05,−0.20]	*d_*s*_* = 0.14 [−0.28, 0.57]
**Manipulation of gender team composition**								
Ryan et al. (2012)	M	F & M	Social support	Male participant—Male Candidate	All-male Composition vs. Balanced Composition	Prime Condition.No differences between all-male and balanced composition. Control Condition. Less social support in all-male composition.	*d_*s*_* = 0.10 [−0.62, 0.83]	*d_*s*_* = −0.39 [−1.17, 0.38]
				Female participant—Female candidate	All-male composition vs. Balanced composition	Prime Condition.No differences between all-male and balanced composition. Control Condition. Less social support in all-male composition.	*d_*s*_* = −0.25 [−0.80, 0.31]	*d_*s*_* = −0.52 [−1.08, 0.04]
			Professional Evaluation	Male participant—Male Candidate	All-male composition vs. Balanced composition	Prime Condition.No differences between all-male and balanced composition. Control Condition. Higher positive evaluation in all-male composition.	*d_*s*_* = 0.03 [−0.69, 0.75]	*d_*s*_* = 1.02 [0.22, 1.82]
				Female participant – Female Candidate	All-male Composition vs Balanced Composition	Prime Condition. Higher positive evaluation in all-male composition. Control Condition. No differences between all-male and balanced composition.	*d_*s*_* = 0.59 [0.02, 1.16]	*d_*s*_* = −0.17 [−0.72, 0.39]
**Manipulation of discrimination - high status (i.e., white male) discriminated target**								
Wilkins et al. (2013)	M	White male	Positive evaluations	Anti-white bias claim vs. No claim		When exposed to claim of anti-male bias, the high status target is evaluated more positively in the prime condition than in the control condition.	*d_*s*_* = 0.52 [0.19, 0.86]	*d_*s*_* = -0.35 [−0.69, −0.02]
						When exposed to no claim, the high status target is evaluated less positively in the prime condition than in the control condition.		
			Helping intentions			When exposed to claim ofanti-male bias, the intentions of helping the discriminated high status target are higher in the prime condition than in the control condition.	*d_*s*_* = 0.50 [0.17, 0.83]	*d_*s*_* = −0.16 [−0.49, −0.17]
Wilkins et al. (2017)	M	White female	Target evaluation	All participants were expose to Anti-White Bias Claim		When exposed to claim of anti-male bias, the discriminated low status target evaluated more positively in the prime condition than in the control condition.	*d_*s*_* = −0.97 [−1.49, −0.44]	*d_*s*_* = −0.16 [−0.60, 0.28]
			Helping intentions			When exposed to claim ofanti-male bias, the intentions of helping the discriminated low status target are higher in the prime condition than in the control condition.	*d_*s*_* = −0.70 [−1.21, −0.18]	*d_*s*_* = 0.01 [−0.43, 0.45]
**Manipulation of locus of causality—low status (i.e., female) discriminated target**								
McCoy and Major (2007)[study 2]	M	F	Perceived discrimination	Attribution to: Sexism vs. Control		When the discriminatory behavior is attributed to Sexism, participants in the prime condition, perceived prejudice against the female candidate to a significantly lower degree than in the control condition. In the control condition, there are no differences between prime and control condition.	*d_*s*_* = −3.50 [−4.90,−2.11]	*d_*s*_* = −0.11 [−0.99, 0.77]
			Stereotypes endorsement			When the discriminatory behavior is attributed to Sexism, participants in the prime condition endorse gender stereotypes to a significantly higher degree than in the control condition. Control Condition. No differences between prime and control condition.	*d_*s*_* = 2.52 [1.35, 3.69]	*d_*s*_* = 0.12 [−0.76, 1.00]
Redersdorff et al. (2016)[study 2]	M	F	Perception of Competence	Positive Discrimination (equivalent level vs. minimum required vs. unconditional preference)		When the discriminatory behavior is attributed to Sexism, the discriminated female target is judged less competent in the prime condition in the social equality condition. When the discriminatory behavior is internally attributed (e.g., less ability), the discriminated female target is more competent in the prime condition, than in the control condition.	*d_*s*_* = −0.84 [−1.26, −0.41]	*d_*s*_* = 1.60 [1.13, 2.07]
			Personal accountability			When the discriminatory behavior is attributed to Sexism, the discriminated female target is held less personal responsible in the prime condition than in the social equality condition. When the discriminatory behavior is internally attributed (e.g., less ability), the discriminated female target is held equality responsible across the two conditions.	*d_*s*_* = −3.35 [−3.99, −2.7]	*d_*s*_* = 0.08 [−0.33, 0.49]

*Social Distance* The relationship between the prime and social distance is moderated by individual's levels of PWE. When PWE is high, after being exposed to the PWE- prime condition, the Black Target is judged less favorably (vs. White target). In contrast, when PWE is low, there are no significant status-based differences in prime condition (Biernat et al., 1996).

*Same-Gender Professional Evaluation* Gender team composition was found to moderate the relationship between gender, prime and ingroup evaluations. In groups composed only by males, female participants after being exposed to the high social mobility condition (vs. low social mobility) were more likely to favor the female target (see Table 5). In contrast, in a gender-balanced group, female participants after being exposed to the high social mobility condition (vs. low social mobility) were less likely to favor the female target. A different pattern was found for men. In all-male group composition, male participants, after being exposed to the high social mobility condition (vs. low social mobility) were less likely to favor the male target. In the gender-balanced group, male participants after being exposed to the high social mobility condition (vs. low social mobility) were more likely to favor the male target (Ryan et al., 2012).

**Table 5:** Summary of studies related with the Impact of Meritocracy on Self—Evaluations and Performance.

**Study**	**Outcome**	**VI 1**	**VI 2**	**Results**	**Effect size**	
					**Prime**	**Control**
Quinn and Crocker (1999)	Psychological Well being	Overweight		Overweight women in the prime condition show lower scores than overweight women in the control condition.	*Not Estimable*	
		Normal weight		Prime did not predict differences in PWB.		
	Self-esteem	Overweight		Overweight women in the prime condition show lower scores than overweight women in the control condition.		
		Normal weight		Prime did not predict differences in SE.		
Darnon et al. (2017)	School performance	Low SES vs. High SES		In the prime condition performance was significantly lower for Low SES students than high SES students, compared to control condition.	*d*_s_ = −4.43 [−5.38, −3.48]	*d*_s_ = −0.80 [−1.31, −0.29]
	School efficacy			In the prime condition school self- efficacy was lower for Low SES students than high SES students, compared to control condition.	*d*_s_ = −0.47 [−0.99, 0.05]	*d*_s_ = –0.12 [−0.60, 0.37]
McCoy and Major (2007)[study 1]	Attributions for rejection	Women	Discrimination vs. Internal attributions	In the prime condition, women were more likely to make internal attributions for the rejection (e.g., blame themselves) than blame on discrimination. Women in the control condition were no more likely to blame themselves than they were to blame discrimination	*d*_z_ = −0.44	*d*_z_ = −0.18
		Men	Discrimination vs. Internal attributions	In the prime condition, men were no more likely to blame themselves than they were to blame discrimination. in the control condition were more likely to blame themselves than they were to blame discrimination.	*d*_z_ = −0.07	*d*_z_ = 0.70

##### Does Meritocracy predict less favorable self-evaluations, internal attributions, and poorer performance?

Table 5 shows that priming Meritocracy increases negative self-evaluations, internal negative attributions and decreases school performance in low-status and stigmatized group members.

*Self-evaluations* When primed with Meritocracy, overweight women showed lower psychological well-being as well as lower self-esteem than overweight women in the control condition. Normal weight women did not show significant differences in psychological well-being or self-esteem between conditions (Quinn and Crocker, 1999).

*School Performance* In an educational context, primed low socioeconomic students (SES) performed significantly lower in a French and Math performance test than high socioeconomic students, compared to low and high socioeconomic students in the control condition (Darnon et al., 2017). In the prime condition, low SES students did not show significantly lower self-efficacy than high SES students, compared to the control condition.

*Locus of Causality* Women primed with Meritocracy were more likely to make internal attributions for the rejection (e.g., blame themselves) than to blame it on discrimination, while in the control conditions women were not more likely to blame themselves than they were to blame discrimination. In contrast, men primed with Meritocracy were not more likely to blame themselves than they were to blame discrimination. Interestingly, men in the control condition show an opposite pattern: they were more likely to make internal attributions for the rejection (e.g., blame themselves) than to blame it on discrimination (McCoy and Major, 2007; study 1).

##### Does Meritocracy predict less favorable decisions toward low-status groups?

*Adhesion to the European Union* A single study found a small effect of Meritocracy predicting opposition to Turkish Adhesion to the EU (vs. control condition; Pereira et al., 2009).

*Monetary rewards* Across four studies conducted in the US, a less favorable outcome for the female target (vs. male target) was found in the Meritocracy prime condition compared to a control condition (Castilla and Benard, 2010; Thomson, 2015). The female candidate was consistently less rewarded (e.g., bonus reward), compared to the equally qualified male candidate, when priming participants with Meritocracy (*vs*. control condition). Other types of decisions (e.g., hiring or promotion decisions) were not impacted by the prime condition (see Table 6).

**Table 6:** Summary of studies related with the Impact of Meritocracy and Decisions toward Low and High Status Targets.

**Study**	**Target**	**Outcome (Low status—High status)**	**Results**	**Effect size d [95% IC]**	
				**Prime**	**Control**
Castilla and Benard (2010) [study 1]	Women	Monetary reward	Less favorable outcome for female target in the prime condition	d_z_ = −0.31	d_z_ = 0.27
		Hiring decision	No differences between female and male target.	d_z_ = 0.00	d_z_ = 0.02
		Promotion decision	No differences between female and male target	d_z_ = −0.08	d_z_ = 0.09
Castilla and Benard (2010) [study 2]	Women	Monetary reward	Less favorable outcome for female target in the prime condition	*Not estimable*	*Not estimable*
Castilla and Benard (2010) [study 3]	Women	Monetary reward	Less favorable outcome for female target in the prime condition	d_z_ = −0.30	d_z_ = 0.01
Thomson (2015)	Women	Monetary reward	Less favorable outcome for female target in the prime condition	*d*_s_ = −1.69 [−2.04, −1.35]	*d*_s_ = −0.47 [−0.79, −0.15]
Moreira (2016) [study 1]	Homeless	Acceptability of sacrificing the target	No differences between homeless and White male target	*d*_s_ = −0.51 [−0.79, −0.15]	*d*_s_ = −0.57 [−0.84, −0.29]
Moreira (2016) [study 2]	Homeless		Less acceptability of sacrificing the low target, relative to the high status target the in the prime condition.	*d*_s_ = −0.50 [−1.09, 0.08]	*d*_s_ = 0.14 [−0.42, 0.70]
	Drug addict		No differences between drugs addict target and White male target in the prime condition.	*d*_s_ = 0.0 [−0.57, 0.57]	*d*_s_ = −0.32 [−0.89, 0.24]

*Acceptability of sacrificing the target* In a trolley dilemma, priming Meritocracy made the decision to sacrifice a low-status target (i.e., drug addicts) for the sake of saving five individuals more acceptable (vs. the control condition). This result was not found with another type of low-status target (i.e., homeless; Moreira, 2016). Moreover, when comparing asymmetrical targets, priming Meritocracy made the decision to sacrifice a low target (e.g., homeless) less acceptable than sacrificing a high-status target (e.g., White male), whereas, sacrificing a drug addict was equally acceptable as sacrificing a high-status target (e.g., White male).

*Monetary donation* Donation to a homeless shelter was found to be moderated by the way PWE prime was induced. Manipulating the *justifier of inequality meaning* of PWE decreased the likelihood of donating money to a homeless shelter (vs. manipulating the *social equalizer meaning* of PWE) (Levy et al., 2006, *study 4*).

## Summary of the Main Findings and Discussion

The impetus for initiating this systematic review was the number of mixed results across studies in the literature, including inconsistent findings from our own lab that used Meritocracy prime paradigms to test status-based differential outcomes. In some of our experiments, for example, we found that Meritocracy beliefs positively predicting outcomes toward the low-status target; in other experiments, we found Meritocracy beliefs negatively predicting outcomes toward the low-status target. Thus, the purpose of this systematic review was to (a) analyze whether priming Meritocracy predicted less favorable outcomes toward low status groups; (b)summarize the content of the different prime tasks, and (c)summarize prime manipulation checks' effectiveness.

The present systematic review examined 33 studies that contained 62 outcomes. The work we reviewed was distributed across six domains and spanned 29 years of research. Results across studies revealed that despite the existing differences in the components highlighted, the salience of any of the Meritocracy dimensions facilitates the use of internal causal attributions, negative evaluations, and stereotyping toward low status groups, affecting negatively decisions involving low-status group members, particularly in organizational contexts.

Our analysis helps identify basic components of Meritocracy beliefs and systems and illuminate how these components are organized and framed within the scope of other satellite concepts. We have shown that both Meritocracy and PWE primes incorporate the *effort/hard work* aspect of both ideologies. In addition to this aspect, Meritocracy primes, to a large extent, incorporate internal locus of control, and to some extent the idea that people can achieve social mobility. In turn, PWE prime captures, to a smaller extent, the *internal control* aspect, comparing to the Meritocracy prime. Additionally, social mobility primes fully focus on this structural component of Meritocracy beliefs. And finally, a core dimension that is largely absent in most of the primes is the idea that the social system provides equal opportunities for all, with the exception of two studies (Castilla and Benard, 2010; Darnon et al., 2017).

Although differences in the degree to which the dimensions are present may vary, the patterns we observed seem to suggest that the two internal dimensions that inform the concept of Meritocracy—*effort/hard work* and *internal control*—are, to a large extent, present both in Meritocracy and PWE primes. In other words, the Meritocracy and PWE primes show similarities by integrating those internal dimensions. In turn, Meritocracy and social mobility primes show similarities by integrating, to a greater extent, the external dimensions, as for example the *social mobility* aspect.

Regarding the type of meritocracy used in the tasks of inducing meritocracy, a large number of studies mixed both prescriptive and descriptive components of meritocracy or present an unspecified meritocracy content; only six of the studies focused on the descriptive component of meritocracy. As previously discussed by Son Hing et al. (2011), believing that a system is meritocratic (descriptive) is different from thinking it should be meritocratic (prescriptive). While descriptive meritocracy is argued to be negatively related with intergroup outcomes, prescriptive meritocracy is not. Although it is not possible to determine to what extent this factor explains why some studies fail to produce the desired effect, it is possible that when the two types of meritocracy (prescriptive and descriptive) are salient in a task, the way the effect of meritocracy will operate may depend on the interpretation made by the participant, which in turn, may apply either the social equalizer or the social justifier function, depending on the context and nature of the study.

Concerning the prime affecting the activation of the concept, a substantial number of studies did not report a prime manipulation check. As a result, we were unable to assess the effectiveness of those experiments. Among those studies measuring the level of endorsement, Meritocracy prime paradigms reported higher endorsement of meritocratic beliefs, compared to PWE prime paradigms. However, this conclusion should be interpreted with caution since there is high variability between Meritocracy and PWE primes.

As far as our systematic search could find, the original paradigm developed by McCoy and Major (2007) was used in 6 subsequent studies, across four countries (US, Canada, Netherlands, and Portugal). Despite being the most frequently implicit task used, only two single manipulation checks were reported, suggesting primed individuals more strongly agree about individual mobility (e.g., America is an open society in which success is possible for all individuals; McCoy and Major, 2007), are more optimistic about the future of societal fairness and strongly agree about societal justice in the country (e.g., Canada; Laurin et al., 2011). These findings suggest that a subtle activation temporarily increased not only specific aspects of Meritocracy value but also broader aspects associated with fairness and satisfaction with the social system (Jost and Hunyady, 2005; McCoy et al., 2013). This way, when the content of the prime reflects alone features of a Meritocracy system, individuals tend to perceive that system as a more permeable one, in which, through hard work and talent, people can move individually into a higher social position. This perception of a greater status permeability within the system is reflected in a greater belief in societal fairness and social equality. Such version of Meritocracy beliefs can be found in popular “rags to riches” stories, with the implication that people from all social categories have equal potential to succeed through hard work and effort. So, this evidence is consistent with the meaning of Meritocracy as a *social equalizer*, as defined by Levy and colleagues, and associated with greater egalitarianism (see Levy et al., 2005, 2006, 2010) across different age and social status groups (Levy et al., 2006).

But when people are made to believe that meritocracy exists in the social system (Son Hing et al., 2011), and subsequently are presented with information that contradicts that same social reality, then is likely that individuals engage in justifications to explain the dissonance about how the system should be, but is not. So, when a system is not meritocratic but people believe that it is a Meritocracy, such a mismatch, it is likely to have significant social implications for intergroup relations. For example, members of low status groups may be inclined to see their social position as legitimate and thus be accepting, and high status group members may logically infer low status groups as individually responsible for their disadvantage position in the social system (McCoy and Major, 2007; Rüsch et al., 2010). This legitimizing intergroup dynamics makes the role of Meritocracy beliefs fundamental to understand the maintenance of social inequality. Particularly, in social systems characterized by asymmetrical status relations (Jost et al., 2003). In such societies, Meritocracy as a socially shared system of beliefs, serves as a social glue, holding the status-based hierarchy, and importantly, making inequalities more acceptable (Jost and Hunyady, 2003; Son Hing et al., 2011; Major and Kaiser, 2017), hence promoting stability within stratified social system (Tajfel and Turner, 1979; Kay and Friesen, 2011; Laurin et al., 2013).

Moreover, in such societies, Meritocracy beliefs seems to operate as a facilitator of intolerance toward low status groups, by rendering access to attributional, stereotypical and negative inferences about specific social groups (Biernat et al., 1996). As the combining results show, participants more easily show implicit negative attitudes, infer negative internal attributions and stereotyping, after being primed (vs. control group; Ho et al., 2002; Costa-Lopes et al., 2017).

In addition, it has consequences for preserving the *status quo* of dominant groups in asymmetrical contexts (Jost et al., 2003). And how? For example, by opposing policies aimed at promoting greater equality between groups (Wellman et al., 2016) or by decreasing egalitarian values in adults (but not young children; Levy et al., 2006, *study 3*). However, this is true only when Meritocracy is used as a *justifier* (Levy et al., 2006), and is more likely to be a justifier when it is also perceived to be a *descriptive* social system (Son Hing et al., 2011). And that is because when prescriptive, Meritocracy favors acceptance of merit-upholding social policies designed to bring about more intergroup equality in the workplace (e.g., positive discrimination) and more opposition to a merit-violating policy (Chatard et al., 2006). This evidence is in line with correlational studies on principled Meritocracy suggesting that people with stronger prescriptive beliefs about merit were more opposed to merit-violating policies but not more opposed to merit-upholding program policies than were people who weakly endorsed the merit principle (Bobocel et al., 1998; Davey et al., 1999).

Moreover, the idea that priming Meritocracy leads to more negative evaluations of low status after exposing participants to claims of anti-white bias (Wilkins et al., 2018), can also be understood within a broader conceptual framework involving social identity theory. From a social identity perspective (Turner et al., 1979), exposing the high-status groups to a type of claim (e.g., anti-male bias) portraying a threat (e.g., discrimination) could make salient certain social categories and potentially threaten the identity of those individuals who belong to high-status groups. If this is correct, it would result in high ingroup identification for high-status group members (but not necessarily for low-status group members; see Wilkins et al., 2017) and in ingroup favoritism as shown by the increased positive evaluations and helping intentions toward a White male target found by Wilkins et al. (2013), and in outgroup derogation (Wilkins et al., 2018). The fact that this result happens only when Meritocracy is salient suggests that as a socially shared system of beliefs, Meritocracy opens the door to support for high status group members when their identities are threatened.

Finally, when causal attributions are added to the picture, it serves to accredit the target with more or less value (Pansu et al., 2003). In order for Meritocracy to perform its function of providing psychological comfort (Jost et al., 2008; Bahamondes et al., 2019), it is necessary to convey the idea of individual control and responsibility over the (lack of) success achieved or the belief in a socially mobile society (Sagioglou et al., 2018). Because when others' failure occurs, it is cognitively easier to attribute it to internal rather than external explanations (Ross, 1977), Meritocracy beliefs, through the work of its internal hard drives—effort/hard work and internal control—acts as a facilitator of internal explanations to decrease the perceptions of group-based discrimination. For example, telling participants that a female target has been discriminated against due to sexism led participants (exposed to the prime) to perceive less prejudice against the female candidate, to endorse gender stereotypes to a significantly higher degree (McCoy and Major, 2007) and to judge the female target as less competent (Redersdorff et al., 2016). Because attributing a discriminatory result to external causes invites us to challenge the legitimacy of this discrimination, it is necessary to neutralize potential threats that call into question the legitimacy of such discrimination. To this end, activating in people's minds Meritocracy beliefs facilitates the access to stereotypical inferences and evaluations, which in turn, are used to neutralize gender-based discrimination perception in the workplace. These results are aligned with recent findings suggesting that individuals show a greater tendency to engage in meritocratic beliefs and support inequality (Brandt, 2013; Kraus and Callaghan, 2014; Kraus et al., 2017).

Interestingly, telling female participants that a female target has been discriminated against due to internal factors (e.g., less competence) led merit-primed participants (vs. social equality-primed) to see the ingroup target as more competent (Redersdorff et al., 2016). This finding is striking given the fact that Meritocracy -primed females react more favorably toward the ingroup target when the behavior does not challenge the legitimacy of the negative outcome. As if attributing a discriminatory outcome to internal causes (e.g., lack of competence) is to postulate the legitimacy of the negative outcome. As such, this finding is relevant for studies shedding light into the interplay of Meritocracy and internality norms because it opens new ways through which the legitimacy of inequality may operate (Walker, 2014).

However, what are the limits to this palliative effect that underlies the meritocratic ideology? For disadvantaged groups, one of the consequences shown is that individuals primed with Meritocracy showed a lower psychological well-being and lower self-esteem, especially marginalized groups, such as overweight women (Quinn and Crocker, 1999). Another example arises in the educational context, where, primed low SES students performed significantly lower in a performance test than high SES students, compared to students in the control group (Darnon et al., 2017). Interestingly, among students, self-efficacy had a buffering effect, as the strength of the causal relationship between Meritocracy and school performance decreased after taking into account self-efficacy. A third finding suggests that if people are led to believe that Meritocracy exists, in the face of discrimination or failure, they are more prone to blame themselves than the system. And this is particularly true for members of low status groups (McCoy and Major, 2007). This evidence is consistent with the idea developed by Jost and colleagues that the system-justifying beliefs serve the palliative function of reducing the negative effect of an unfavorable situation, especially, but not exclusively, among low-status groups (e.g., Jost and Hunyady, 2003).

### Limitations

The present research focused exclusively on experimental studies developed with the aim of testing whether by priming Meritocracy beliefs, low status groups would be more socially disadvantaged in a range of outcomes. We made this decision because our focus is (a) on the consequences of Meritocracy only for low status groups and (b) on the causal inference between the two variables. Our goal in the present review was to stimulate a more systematic study of how Meritocracy primes affect the activation of concepts associated with Meritocracy beliefs, which in turn affect social perceptions and behaviors. Because of the still limited body of experimental research on this topic and the broad range of ways that Meritocracy has been conceptualized, we pursued a systematic review. However, as the body of work on this topic grows and becomes more conceptually cohesive, a formal meta-analytic review may complement our initial analysis in important and empirically fertile ways.

As with all reviews, this assessment is vulnerable to publication bias despite our extensive search for unpublished and ongoing experimental studies. Although our search for unpublished literature extended to January 2019 we could only include three unpublished experiments. Furthermore, the sample size of the analyzed studies is generally small, given the new standards in psychological research. As a result, more research should be conducted on larger samples in order to draw clearer conclusions on the effect of meritocracy manipulations on intergroup outcomes.

Of importance, a substantial number of studies did not report a prime manipulation, making it largely unfeasible for us to compare the effectiveness of manipulation in activating the concept. The question of whether manipulation checks are necessary is currently under discussion (see Fayant et al., 2017), but a more precise, cumulative estimate asserting internal and construct validity of the prime would benefit the research (Foschi, 2014; Flake et al., 2017).

The studies included in our systematic review were carried out predominantly in the United States. The search strategy uncovered a few studies from European countries, so some caution is needed when assuming that the results found mostly in one country, apply equally to other nations. For example, researchers have noted that acceptance of inequality is informed by the levels of inequality people perceive to exist in the country (Castillo, 2012; Trump, 2017). Moreover, acceptance of inequality and desire for inequality are stronger for highly meritocratic individuals and in countries were meritocratic norms are more salient (e.g., the United States; García-Sánchez et al., 2018). So, the general detrimental effects of Meritocracy beliefs for low status groups found in this systematic review should be considered to apply to a social context where Meritocracy is more salient. Because, as noted in other studies, in less status permeable countries where meritocracy beliefs are less salient, Meritocracy beliefs are less likely to acquire the legitimizing function and instead operate through its socially equalizer mechanism (Crandall and Martinez, 1996; Ramírez et al., 2010; Rosenthal et al., 2011). Hence, further published and unpublished research outside the United States would benefit the field. Particularly, in countries were perceptions of upward social mobility are lower, but work ethic beliefs are high.

Finally, we were able to calculate the size of the control and treatment group effect for most variables at the target level, but calculating the combined effect of the different primes was beyond our purpose. Thus, the results we described are limited to the effect of each treatment or control group on the outcome of interest.

## Conclusions

Understanding the adverse social consequences of Meritocracy beliefs for disadvantaged groups is clearly important, especially when inequality across western societies is continuously rising (Organisation for Economic Co-operation Development, 2016; World Bank, 2016). The results found in this study derived from a two-stage process carried out to explore the possibility of integrating Meritocracy, SLBs and PWE effects on socially relevant outcomes involving low status groups, while systematizing the commonalities among the various paradigms currently used. Although Meritocracy, SLBs and PWE prime show differences in the components highlighted, these differences seem to produce similar results. That is a confirmation of the general hypothesis according to which low status groups members are more likely to receive an unfavorable outcome when Meritocracy (or similar) is made salient, compared to any other experimental or control condition. The salience of any of the components of Meritocracy beliefs facilitates the use of internal causal attributions, negative evaluations and stereotyping toward low status groups, affecting negatively, decisions involving low-status group members, particularly in some domains (e.g., organizational). Moreover, the way in which Meritocracy beliefs seems to operate is key for producing the social glue necessary for the stability of whatever inequalities.

The findings also give a hint on how the components of Meritocracy beliefs may be informing the way lay people justify why low-status individuals are more likely to be discriminated against, by for example facilitating the access to stereotypical and attributional content about others (e.g., *effort/hard work* aspect); to offer psychological comfort, by conveying the idea of individual responsibility to promote a feeling of greater control over the environment (e.g., *internal control* aspect); and finally to promote a stable social system between asymmetric groups, by conveying the idea of equal opportunities and upward social mobility for all, an idea highly valued in western societies (e.g., *social mobility and equal opportunities* aspect).

Ideally, this work will inform and facilitate further research aimed at understanding when and under which circumstances the belief in Meritocracy disproportionally affects members of relative disadvantaged groups, and how each component may be used to perpetuate the existing evidence concerning the negative consequences for intergroup relations. By doing so we may gain a better understanding of how to tackle the downside of the belief in Meritocracy.

## Data Availability

The datasets analyzed in this manuscript are not publicly available. Requests to access the datasets should be directed to filipa.madeira@ics.ulisboa.pt.

## Author Contributions

AM designed the study and was responsible for writing the initial and final version of the paper. GF and MM collected the data, searched for references, and performed data extraction. RC-L supervised the study at all stages and contributed to the writing of the final version of the paper. JD supervised and contributed during the paper writing process.

### Conflict of Interest Statement

The authors declare that the research was conducted in the absence of any commercial or financial relationships that could be construed as a potential conflict of interest.

## Footnotes

^1^We use the term Meritocracy primes here to refer to experimental manipulations of meritocracy and similar constructs cues that seek to activate the belief in Meritocracy. These may be consciously recognized (explicit primes) or unconsciously perceived (implicit primes).

^2^Also the reason for the use of the term Meritocracy primes along the paper.

^3^Veritas Health Innovation, Melbourne, Australia. Available online at www.covidence.org
