# Supplementary material for: Primes and Consequences: A Systematic Review of Meritocracy in Intergroup Relations
Source: Front Psychol. 2019 Sep 19;10:2007. doi: 10.3389/fpsyg.2019.02007 (PMC6761281; doi:10.3389/fpsyg.2019.02007)
Supplement: Supplementary file 1 [file Data_Sheet_1.PDF]

Table S1

Descriptive Summary of Characteristics and Construct Salience

Study	Task Description	Dimensions of Prime: Effort/ hard-work	Internal Control	Social Mobility	Equal Opportunities	Type of Task	Manipulation Check
**Construct : Meritocracy**							
Castilla et al. (2010)	**Prime condition -** (1) "All employees are to be rewarded fairly"; (2) "whether employees deserve a raise is determined by their performance"; (3) "raises and bonuses are based entirely on the performance of the employee"; (4) "promotions are given to employees when their performance shows that they deserve it"; (5) "ServiceOne's goal is to reward all employees equitably every year." **Other condition -** (1) "All employees are to be evaluated regularly"; (2) "whether an employee deserves a raise is determined by their manager"; (3) "raises and bonuses are to be given based on the discretion of the manager"; (4) "promotions are to be given to employees when their manager decides that they deserve it"; and (5) "ServiceOne's goal is to evaluate all employees every year."	**Fully**	Not at All	Not at All	**Fully**	Explicit	Yes
Chatard et al. (2006)	**Prime Condition:** Meritocracy : moderate "autant que possible" **Other Condition:** Meritocracy : radical "systématiquement" 1. Dans la vie, les gens doivent ***autant que possible/ systématiquement*** obtenir ce qu'ils méritent . 2. Les augmentations de salaire doivent ***autant que possible/ systématiquement*** être proportionnelles aux efforts des salaries. 3 Dans les entreprises, les salariés qui travaillent mieux que les autres doivent ***autant que possible/ systématiquement*** avoir un meilleur salaire.	**Fully**	Not at All	Not at All	Not at All	Explicit	Yes
Costa-Lopes et al (2017) [1]	Adapted from Pereira et al. (2009)	**Largely**	Somewhat	**Largely**	Not at All	explicit	No
Costa-Lopes et al (2017) [2]	Adapted from McCoy et al. (2007)	**Fully**	**Largely**	**Largely**	Not at All	Implicit	No
Darnon et al. (2017)	**Prime Condition** - 'At school, everyone has the same chances to begin with, but only some students manage to succeed: those who have the ability and motivation. Indeed, to succeed in school, children have to deserve it. (. . .). Thus, if students fail, they can only blame themselves. To succeed in school, one just has to work well; it is only a matter of will'. **Other Condition** - neutral text dealing with frogs' ability to anticipate disasters was presented. Children were asked to summarize the content in one sentence.	**Fully**	**Fully**	Somewhat	**Fully**	Explicit	Yes

Darnon et al. (2018)	**Prime Condition** - "At school, where there is a will, there is a way" and argued that research has fully documented that motivation, will, and hard work are the main ingredients of school success"<br>**Other Condition** - neutral text defending the idea that the way a backyard space is organized can determine children's games and did not mention merit.	**Fully**	**Fully**	Somewhat	**Fully**	Explicit	Yes
Ho et al (2002) [1]	**Prime condition** participants watched videotaped segments from the Horatio Alger awards program. The focus of the program was the award winners' bleak beginnings, the obstacles that they had to overcome, and the qualities that they possessed that enabled them to succeed.<br>**Other Condition**: participants watched videotaped segments from a PBS program on animal antics.	**Largely**	**Largely**	**Fully**	Not at All	Explicit	Yes
Moreira (2016)	**Prime condition** - Lazy people don't succeed; Those who make an effort succeed; Unsuccessful people are lazy; Those who work the most receive; If you've worked, you've got a lot of money; Who's bad doesn't work; Only the best get away with it; The rewards depend on the effort; Wealth results from work; We must all strive.<br>**Other condition** - We just got back from shopping; Lisbon is a European city; The peasant flowers are fresh; Do not forget your coat. To read something is to travel still; Using a calculator saves time; Birds fly too high; Time goes by too fast; The night is a good counsellor; She likes to cook fish; He had a good idea; Television has many channels; When it rains she wears a hat; She likes cats a lot; Gravity pulls objects; Geniuses think alike; The surface is very smooth; I think it's going to rain tomorrow."	**Fully**	**Largely**	Not at All	Not at All	Implicit	No
McCoy et al. (2007)	**Prime condition** – independent people do well, accomplishing goals is satisfying, usually diligence is rewarded; usually life is fair ; judge people on merit; earn a good living; ambition moves you forward; effort leads to prosperity; competition is very healthy; responsible people get ahead; persistence leads to success; encourage children to dream; hard work is effective; self-reliance makes you strong; rich people deserve it;<br>**Control condition** - always wear your hat; a calculator saves time; exercise can be fun; drinks a lot of water; keep your hands clean	**Fully**	**Largely**	**Largely**	Not at All	Implicit	Yes
Pereira et al (2009)	**Prime condition:** ''In current societies, there is increasing demand in professional relations for people to have the type of training that highlights the values of merit, such as competence and productivity in their competitive ability".<br>Following 2 items taken from Katz and Hass (1988).<br>**Other condition:** ''In current societies, characterized by the attempt to promote equality, professional relations increasingly demand that people should have the type of training that really enhances their egalitarian values, such as equality and social justice".	**Largely**	Somewhat	**Largely**	Not at All	Explicit	Yes

Redesdorff et al. (2016)	**Prime condition** - participants were informed that the six events were symbolic of personal merit and that anything is possible when people give it their all (or, as in the old saying, 'Where there's a will, there's a way'; e.g., Harry Roselmack and his arrival on French TV as the first black newscaster).<br>**Other Condition** - six events were symbolic of social equality and the struggle against social injustice (e.g., Gandhi and the Salt March).<br>Task 2 - search-word puzzle.<br>This puzzle constituted of a 21 x 21 matrix of letters in which 14 words were written horizontally or vertically. The participants were asked to identify the most words they could find in 10 minutes. They were informed that each word contained at least six letters. The matrix included seven words related to meritocratic values (e.g., effort), and seven words related to equality values (e.g., equality).	**Fully**	Not at All	Not at All	Not at All	Explicit	Yes
Thomson et al. (2015)	**Prime condition**: (1) All employees will be rewarded on a fair basis. (2) Bonuses and raises are completely based on the performance of the employee. (3) The performance from the employee will determine whether they deserve a raise; (4) Promotions will only be given to employees who show they have earned it through their performance at work; (5) Moyes Insurance Group will reward all employees equitably every year.<br>**Other condition:** (1)All employees are to be evaluated regularly; (2) Raises and bonuses will be given yearly to employees who remain their tenure with the organization; (3) Promotions given to employees' will be decided by the discretion of the manager; (4) Whether an employee deserves a raise is to be determined by their manager; (5) Moyes Insurance group's goal is to evaluate all employees every year.	**Fully**	Not at All	Not at All	**Fully**	Explicit	Yes
Laurin et al (2011) [4]	Adapted from McCoy et al. (2007)	**Fully**	**Largely**	**Largely**	Not at All	Implicit	Yes
Laurin et al (2011) [6]	**Prime condition**: Adapted from McCoy et al. (2007)<br>**Control condition**: groups face cat disadvantages some; coat society be unequal can; hat always seatbelt wear your; hard work sound enough isn't; difficult is often achievement movies; often unfair is coffee life; hardships face many an people; wise the are people elderly; the advance to it's hard; can people biased be fork; life others make cup difficult; for are opportunities not equal<br>rewards given should unfairly are; people good get anyway punished; beautiful paintings lead can be; success people few idea achieve; fly bugs light end towards; blocks opportunities personal often discrimination, sometimes people bad very succeed; hard often glass fail workers.	**Fully**	**Largely**	**Largely**	Not at All	Implicit	No
Wellman et al (2016)	Adapted from McCoy et al. (2007)	**Fully**	**Largely**	**Largely**	Not at All	Implicit	No
Wilkins et al (2013)	Adapted from McCoy et al. (2007)	**Fully**	**Largely**	**Largely**	Not at All	Implicit	No
Wilkins et al (2017)	Adapted from McCoy et al. (2007)	**Fully**	**Largely**	**Largely**	Not at All	Implicit	No
Construct: Protestat ethic Work							

Biernat et al. (1996) [2]	**Prime Condition** ''. . . men and women . . . ought to take pride in their work, work hard, produce as much as possible, and earn an honest day's wage for an honest day's work. . . . Those who are able to work but do not, drain the economic power of a great society by taking the product and profit of the hard work and toil of others with little thought to their own contribution. These individuals diminish their own self-worth and tarnish the ethic according to which the productive members of that society live.''<br>**Other Condition** - ''Principles of a rational society would insist that all social inequalities are unnecessary, and unjustifiable, and ought to be eliminated, and that all people are equal in intrinsic value, inherent worth, and essential nature. . . . All people are to be treated as equals . . . simply because they are human. Everyone, no matter what their social status, has rights . . . including equal access to legal and constitutional protection, equal access to service, and also equality of opportunity, and freedom.''	**Fully**	Not at All	Not at All	Not at All	Explicit	Yes, but atypical.
Levy et al. (2006) [2]	**Prime Condition** article describing a report concluding that 'people who work hard do well and have a successful life'<br>**Other Condition** : article describing a report concluding that 'people who work hard are not always successful'.	**Fully**	Unclear	Unclear	Unclear	Explicit	Yes
Levy et al. (2006) [3]	**Prime + Justification condition** - participants were asked to think and write about instances of others using 'people who work hard succeed' in support of their arguments.<br>**Prime + Definition condition** - participants were asked to think and write about what 'people who work hard succeed' means.	**Fully**	Unclear	Somewhat	Unclear	Explicit	Yes
Quinn & Crocker (1999)	**Prime Condition**: America is a country where people can stand proud on their accomplishments. A place where people are free to live and to achieve to their highest potential. Self-reliance and self-discipline are the cornerstones of this country. Perhaps one of the most important beliefs we can hold is the unwavering notion that each person controls his or her own outcomes. We do not blame others for our failures. Instead each person is responsible for his or her own rewards and punishments. Those who are willing to work hard towards their goals have an excellent chance of succeeding. Only the lazy and the unwilling will be unable to meet the goals they set out for themselves. All who strive for perfection should be commended. No persons will blame others for their problems. Instead, we will all accept the responsibility for our flaws as we also accept the responsibility and praise for our accomplishments. Only in these ways can we assert that we have truly endeavored to be our personal best.<br>**Other Condition** America is a country in which we strive to combine our differences into unity. It is a country that is not only rich in opportunities for the individual but also for families and vibrant neighborhoods. A country whose divergent but harmonizing communities are a reflection of deeper community values. The most exciting revolution ever known to humankind began with these three simple words: "We the People. . ." the revolutionary notion that the people grant government its rights, and not the other way around. Just as those who created this Republic pledged to each other their lives, their fortunes, and their sacred honor, so, too, America's leaders today must pledge to each other that we will keep foremost in our hearts and minds not what is best for ourselves or for our party, but what is best for America. In the spirit of Jefferson, let us affirm	**Fully**	**Fully**	Not at All	Not at All	Explicit	Yes

	that, we must work as though there are no Republicans, no Democrats, just Americans. Yes we will have our differences, but let us always remember what unites us far outweighs whatever divides us.						
Newsom (2014)	Adapted from Quinn et al (1999)	**Fully**	**Fully**	Not at All	Not at All	Explicit	Yes
Katz & Hass (1988) [2]	**Prime Condition –** Completion of Protestant Ethic Scale:<br>1. Most people spend too much time in unprofitable amusements. 2. Our society would have fewer problems if people had less leisure time.3. Money acquired easily is usually spent unwisely.4. Most people who don't succeed in life are just plain lazy.3. Anyone who is willing and able to work hard has a good chance of succeeding. 6. People who fail at a job have usually not tried hard enough. 7. Life would have very little meaning if we never had to surfer. 8. The person who can approach an unpleasant task with enthusiasmis the person who gets ahead. 9. If people work hard enough they are likely to make a good life for themselves. 10. I feel uneasy when there is little work for me to do. 11. A distaste for hard work usually reflects a weakness of character.<br>**Other Condition** - Completion of Egalitarianism Scale<br>1.One should be kind to all people. 2. One should find ways to help others less fortunate than oneself.3. A person should be concerned about the well-being of others.4. There should be equality for everyone—because we are all human beings.5. Those who are unable to provide for their basic needs should be helped by others.6. A good society is one in which people feel responsible for one another. 7. Everyone should have an equal chance and an equal say in most things. 8. Acting to protect the rights and interests of other members of the community is a major obligation for all persons. 9. In dealing with criminals the courts should recognize that many are victims of circumstances. 10. Prosperous nations have a moral obligation to share some of their wealth with poor nations.	**Fully**	Somewhat	Somewhat	Not at All	Explicit	No
**Construct : Perceptions of Social Mobility or Economic Success**							
Ho et al (2002) [2]	**Prime condition:** Considerable media attention has been given to the educational and economic attainments of Asian Americans. In recent years, a multitude of national periodicals ranging from *Time* and *Newsweek* to *Psychology Today* have touted "The Triumph of Asian Americans" and labeled Asian Americans students as "The New Whiz Kids". This isn't just media hype. An examination of statistics from a variety of sources confirms that, overall, Asian Americans are thriving in the United States. (…)Given their educational attainments, it is not surprising that Asian Americans are succeeding economically. (…) On the whole, though, Asian Americans are doing well. Through hard work and education, many Asian Americans are succeeding in establishing a place for themselves in the United States.<br>**Other Condition -** Participants read about an archaeological discovery about an early Greek dramatist.	Largely	Not at All	**Fully**	Not at All	Explicit	Yes
Ryan et al (2012)	**Prime condition**: For every 10 participants, there is a slot for 5 people to get into the High Potential Manager group."<br>**Other**: "For every 10 participants, there is a slot for 1 person to get into the High Potential Manager group."	Not at All	Not at All	**Fully**	Not at All	Explicit	Yes

Table S2

Characteristics of the studies included in the systematic review

Experiment	Manuscript Type	Country	Context	Site	Participant's Social Status	Target	Groups	Gender		Age M (*SD*)
								**Female**	**Male**	
Biernat et al (1996)	CH	USA	Uns	LAB	L	~~B~~	2	185		-
Castilla et al., (2010) [1]	JA	USA	Org	CL	L&H	W	2	64	163	29.71 (3.89)
Castilla et al., (2010) [2]	JA	USA	Org	CL	L&H	W	2	45	70	29.29 (4.2)
Castilla et al., (2010) [3]	JA	USA	Org	CL	L&H	W	2	39	62	30 (3.5)
Chatard et al. (2006)	JA	FR	Org	O	L&H	W	6	24	31	39.17 (-)
Costa-Lopes et al (2017) [1]	WP	NL	Uns	LAB	H	I	2	35	7	22.3 (-)
Costa-Lopes et al (2017) [2]	WP	NL	Uns	LAB	H	I	2	28	8	21.2 (-)
Darnon et al. (2018)	JA	FR	Edu	SCH	L&H	L SES	2	80	66	10.13 (0.51)
Ho et al (2002) [1]	JA	USA	Uns	LAB	H	~~B~~	2	97		-
Ho et al (2002) [2]	JA	USA	Uns	LAB	H	M	2	43		-
Katz & Hass (1988)	JA	USA	Uns	LAB	H	~~B~~	2	68	54	-

Laurin et al (2012) [4]	DT	USA	CE	O	L&H	L&H	2	67	24	18.8 (-)
Laurin et al (2012) [6]	DT	USA	CE	O	L&H	L&H	2	73	23	20.4 (-)
Levy et al. (2006) [2]	JA	USA	Uns	SCH	H	NTS	2	276	129	O, 21.31(-) [illegible]
Levy et al. (2006) [3]	JA	USA	Uns	LAB	H	NTS	4	63	72	21.45 (-)
Levy et al. (2006) [4]	JA	USA	SPP	LAB	H	HO	3	67	18[1]	19.96 (-)
McCoy et al. (2007) [pilot [illegible]	JA	USA	Uns	CAM	L&H	NTS	2	13	19	19.56 (1.58)
McCoy & Major (2007) [1]	JA	USA	Org	LAB	L&H	W	4	39	39	19 (1.01)
McCoy & Major (2007) [2]	JA	USA	Org	LAB	H	W	4	41	-	18.6 (.40)
Moreira 2016 [1]	MT	PT	MD	NR	H	HO	4	155	51	22.39 (5.47)
Moreira 2016 [2]	MT	PT	MD	NR	H	HO	6	105	36	25.40 (7.09)
Newsom (2014)	DT	USA	HEA	NR	L&H	NTS	2	201	71	-
Pereira et al (2009)	JA	PT	Uns	NR	H	I	4	20	20	18 – 26
Quinn & Crocker (1999)	JA	USA	HEA	LAB	L	OW	2	118	-	-
Redersdorff et al., 2016 [pilot [illegible]	JA	USA	Org	NR	L	W	2	34	-	32.32 (16.29)

Redersdorff et al., 2016 [1]	JA	USA	Org	STR	H	W	4	95	-	37 (13)
Ryan et al (2012)	JA	USA	Org	O	L&H	W	4	137	96	-
Thomson et al. (2015) [Pilot Study]	MT	USA	Org	O	L&H	NTS	2	37	17	24.3 (-)
Thomson et al. (2015)	MT	USA	Org	O	L	W	2	105	61	23.6 (-)
Wellman et al (2016)	JA	USA	Uns	O	H	MA	2	41	82	32 (9.81)
Wilkins et al (2013)	JA	USA	Org	O	H	MA	2	74	88	30.06 (9.40)
Wilkins et al (2017)	JA	USA	Org	O	H	W	2	-	147	38.32 (13.66)

Note. [1] - 47 unidentified; CH – Book Chapter; JA – Journal Article; DT – Doctoral Thesis; WP – Working Paper; MT – Master Thesis; Uns – Unspecific; Org – Organizational; Edu – Educational; MD – Moral Dilemmas; SPP – Social or Public Policy; CE – Career/Education; HEA – Health; LAB – Laboratory; CL – Class; O – Online; STR – Street; SCH – School; CAM – Campus; L – Low; H – High; B – Black; W – Women; L SES – Low Socioeconomic Status; NTS – No Target specific ;I – Immigrant; M – Mexican American; HO – Homeless; OW – Overweight Women; MA – Male; NR – No data reported. Org – Organizational; Edu – Educational; MD – Moral Dilemmas; SPP – Social or Public Policy; CE – Career/Education; HEA – Health

Table S3

Description of variables used in the Quality Assessment Survey

Variable	Description
**Characteristics at the Study Level**	
study identifier	
type of manuscript	journal article, dissertation, technical report, unpublished manuscript
publication year	
country	
context/ domain of the Study	Educational, health, organizational, social or public policy, unspecific, other
Studies carried	In the lab, Online, on campus, other
Sample's group status	low, high and low and high
Low Status Target	Black, Latinos, Immigrants, Women, No target-specific variable, other.
Study Type	Correlational; Experimental.
Experimental Research Design	between-subjects, within-subjects, mixed design
**Characteristics at the Dependent Variable Level**	
Dependent Variable Assessment	Explicit Measure (e.g. self-report); Implicit measure
Dependent Variable(s) Domain	Self-Attitudinal, Self - Perceptual (Evaluations and/or Judgements), Self - Behavioural, Others -Attitudinal ; Others - Perceptual (Evaluations and/or Judgements); Others – Behavioural
**Studies's Quality Assessment**	

**Fit between Concepts and Operations: Experimental Manipulation**	
Level of detail allowing replication by others	Yes; No
Theoretical Dimensions of the prime: effort/hard-work/ability; internal control/personal; social mobility; racial blindness; equal opportunities	1 – Not at all; 2 – Somewhat; 3 – Largely; 4 – Fully; 9 – Unclear
Manipulation Check	Yes; No
Fit between experimental manipulation description and operationalization	Yes; No
**Fit between Concepts and Operations: Outcome Measure**	
Face Validity	Yes; No
Acceptable Reliability	Yes; No
Outcome measure align with experimental condition	Yes; No
**Clarity of Causal Inference**	
nature of assignment to conditions	random, not-random, not mentioned
Severe Attrition Overall	Yes; No
Experimental and Control groups drawn from the same pool	Yes; No
**Generality of the Findings**	
Sample's sociodemographic variation	Rarely; Largely; Fully
**Precision of Outcome Estimation: Effect sizes and standard errors**	
Outliers Reported	Yes; No
Excluding reason was mentioned	Yes; Maybe; no

Properties of the data allow estimation of the effect sizes	Yes; No
Sample sizes Adequate	Yes; No
**Precision of Outcome Estimation: Statistical Reporting**	
Sample Size Description	Rarely; Largely; Fully
Identification of effects sizes direction	Rarely; Largely; Fully
Effect Sizes Estimation	Rarely; Largely; Fully

Table S4

Appraisal of methodological quality of the experimental studies using DIAD's Items

Experimental studies using DIAD's Items						
	Fit between Concepts and Operations		Clarity of Causal Inference		Precision of Outcomes	
	Experimental Manipulation	Outcome measure	Fair comparison	Lack of contamination	ES and SE	Statistical reporting
Castilla et al., (2010) [1]	Yes	Yes	Maybe no	Maybe no	Yes	Yes
Castilla et al., (2010) [2]	Yes	Yes	Maybe no	Maybe no	No	No
Castilla et al., (2010) [3]	Yes	Yes	Maybe no	Maybe no	Yes	Yes
Chatard et al. (2006)	Yes	Yes	Maybe yes	Yes	Yes	Yes
Costa-Lopes et al (2017) [1]	Maybe yes	Yes	Maybe yes	Yes	Yes	Yes
Costa-Lopes et al (2017) [2]	Maybe yes	Yes	Maybe yes	Yes	Yes	Yes
Darnon et al. (2018)	Yes	Yes	Maybe yes	Yes	Yes	Yes
Ho et al (2002) [1]	Maybe yes	Maybe yes	Maybe yes	Yes	Yes	Maybe Yes

Ho et al (2002) [2]	Maybe no	Maybe yes	Maybe yes	Yes	Maybe Yes	Maybe Yes
Katz & Hass (1988)	Maybe yes	Yes	Maybe yes	Yes	Yes	Yes
Laurin et al (2012) [4]	Maybe yes	Yes	Maybe yes	Yes	Yes	Yes
Laurin et al (2012) [6]	Maybe yes	Yes	Maybe yes	Yes	Maybe yes	Yes
Levy et al. (2006) [2]	Maybe yes	Yes	Maybe yes	Yes	Yes	Yes
Levy et al. (2006) [3]	Maybe yes	Yes	Maybe yes	Yes	Yes	Yes
Levy et al. (2006) [4]	Maybe no	Yes	Maybe yes	Maybe no	Yes	Yes
McCoy et al. (2007) [pilot study]	Maybe yes	Yes	Maybe yes	Yes	Maybe no	Yes
McCoy & Major (2007) [1]	Maybe yes	Yes	Maybe yes	Yes	Maybe yes	Yes
McCoy & Major (2007) [2]	Maybe yes	Yes	Yes	Maybe no	Maybe no	Yes
Moreira (2016) [1]	Yes	Yes	Maybe yes	Yes	Yes	Yes
Moreira (2016) [2]	Yes	Yes	Maybe yes	Yes	Yes	Yes

Newsom (2014)	Maybe yes	Yes	Maybe yes	Yes	Yes	Yes
Pereira et al. (2009)	Yes	Yes	Maybe yes	Maybe no	Yes	Yes
Quinn & Crocker (1999)	Maybe yes	Yes	Maybe yes	Maybe no	Yes	Yes
Redersdorff et al., 2016 [pilot study]	Maybe yes	Yes	Maybe yes	Yes	Yes	Yes
Redersdorff et al., 2016 [2]	Maybe yes	Yes	Maybe yes	Yes	Yes	Yes
Ryan et al (2012)	Maybe no	Yes	Maybe yes	Maybe no	Yes	Yes
Thomson et al. (2015) Pilot study	Maybe yes	Yes	Maybe yes	Yes	Yes	Yes
Thomson et al. (2015)	Maybe no	Yes	Maybe yes	Yes	Yes	Yes
Wellman et al (2016)	Maybe yes	Yes	Yes	Yes	Yes	Yes
Wilkins et al (2013)	Maybe yes	Yes	Yes	Yes	Yes	Yes
Wilkins et al (2017)	Maybe yes	Yes	Yes	Maybe no	Yes	Yes
